# Identification of genetic biomarkers of blood cholesterol levels using whole gene pathogenicity modelling

**DOI:** 10.1007/s00335-025-10140-0

**Published:** 2025-06-06

**Authors:** Sharon Sunny, Guo Cheng, Joshua Haria, Iman Nazari, Jagmohan Chauhan, Sarah Ennis

**Affiliations:** 1 Electronics & Computer Science, University of Southampton, Southampton, SO17 1BJ UK; 2 Faculty Central (Medicine), University of Southampton, Southampton, SO17 1BJ UK; 3 Human Development and Health, University of Southampton, Southampton, SO17 1BJ UK

**Keywords:** GenePy, LDL cholesterol, Statistical analysis, Genetic biomarker identification, Hypercholesterolemia

## Abstract

Elevated cholesterol increases risk of diseases such as heart disease, chronic kidney disease and diabetes and early detection and diagnosis is desirable to enable preventative intervention. This study seeks to elucidate genetic factors affecting low-density lipoprotein cholesterol (LDL-C) levels in blood, enabling development of personalised strategies for lipid management and cardiovascular disease prevention. GenePy, a gene pathogenicity scoring tool, condenses genetic variant data into a single burden score for both individuals and genes. GenePy scores were evaluated across all genes to assess their association with blood cholesterol levels, excluding participants on cholesterol-lowering medications. Nonparametric tests analysed the relationship between GenePy scores and cholesterol levels in those aged < 60 years and ≥ 60 years. GenePy was effective in identifying *PCSK9*, *APOE*, and *LDLR* as the genes most critically influencing plasma cholesterol at a population level. Of note, the strongest genetic effect observed was a *protective* loss of function effect in the *PCSK9* gene. Novel significant signals driving blood LDL-C levels that are common to both age groups include: *BPIFB6* that has a role in lipid binding and transport; *FAIM* that has a role in regulation of lipogenesis, *SLAMF9* previously implicated in macrophage cholesterol loading; *CLU*—a component of HDL; *SAA1* with a known role in cholesterol homeostasis. A gene-based analysis integrating common, rare, and private variations identifies genes influencing blood LDL-C levels. Developing effective polygenic risk scores requires a comprehensive understanding of genetic factors affecting cholesterol to improve prediction and personalise treatment plans.

**Supplementary Information:**

The online version contains supplementary material available at 10.1007/s00335-025-10140-0.

## Introduction

The British Heart Foundation reports that approximately 7.6 million people in the UK, including individuals in all age groups, are living with cardiovascular disease (CVD) (BHF 2024). Although lifestyle factors such as diet and physical activity contribute significantly to CVD risk, genetic predisposition also plays a crucial role. Medical risk factors of CVD include hypertension, diabetes, high cholesterol, and obesity. Analysing blood biomarkers, particularly lipid profiles, is essential for assessing risk and developing therapeutic strategies to prevent cardiovascular events. Elevated cholesterol levels affect 43% of adults in England, and reducing LDL-C by just 1 mmol/L can lower the risk of CVD by 22% (Samarasekera et al. 2023; Trialists et al. 2010). Therefore, maintaining optimal LDL-C levels is critical to mitigating the strong link between high cholesterol and CVD (Chary et al. 2023).

LDL-C levels are stratified into risk categories: concentrations below 3 mmol/L are considered optimal, while levels between 3.0 and 3.3 mmol/L are classified as above optimal. Measurements ranging from 3.4 to 4.1 mmol/L indicate borderline risk, while values between 4.2 and 4.9 mmol/L are categorised as high risk. Levels exceeding 5.0 mmol/L are deemed very high risk, reflecting a significant association with increased CVD incidence (NIH 2024). The most extreme genetic manifestation of increased serum LDL-C level is familial hypercholesterolemia (FH), an autosomal dominant disorder associated with abnormally elevated levels of LDL-C; other conditions such as familial combined hyperlipidemia and polygenic hypercholesterolemia also contribute to hypercholesterolemia. Monogenic FH is primarily caused by pathogenic mutations in *LDLR*, *APOB*, and *PCSK9*, as well as specific variants in *APOE* or rare autosomal variants in *LDLRAP1* (Abifadel and Boileau 2023). Approximately 79% of cases are linked to mutations in *LDLR*, while mutations in *APOB* and gain-of-function (GoF) mutations in *PCSK9* account for 5% and < 1% of cases, respectively. In very rare instances, homozygous recessive FH results from mutations in *LDLRAP1*. Other cases may involve polygenic or monogenic mutations in genes such as *APOE*, *SREBP2*, and *STAP1* (Henderson et al. 2016). A recent cryo-electron microscopy study of LDLR-ApoB100 interaction revealed multiple binding sites and showed how these sites contribute to the overall avidity of the interaction. Mutations affecting this interaction can lead to the development of FH (Reimund et al. 2024).

The genetic basis of hypercholesterolemia exhibits inter-individual variability, influenced by differences in monogenic mutations, polygenic contributions, and their potential interplay with environmental factors. Genetic screening can be conducted to accurately assess which risk factors are relevant to an individual. NHS standard or care now recommends individuals with first-degree family history of FH are referred for cascade testing (Hub 2024). Where a patient’s LDL-C level is above 4.9 mmol/L, or above 4.0 mmol/L and they have a personal or family history of premature atherosclerotic cardiovascular disease, a repeat lipid profile test will be recommended after three months. If, upon follow-up, the individual meets the definition of FH in the Simon Broome, FHWales, or Dutch Lipid Clinic Network criteria, they will be referred for FH genetic testing. If family history is unavailable and the LDL-C level remains elevated despite healthy lifestyle changes, genetic screening will be recommended (Hub 2024).

Patients with moderately elevated lipoprotein levels may benefit from dietary and exercise modifications (Kelly 2010), but those with significantly raised cholesterol typically require medication, primarily statins (Iyen et al. 2021), to manage LDL-C levels. In the UK, around 7.5 million individuals are treated with statins (Kulkarni et al. 2024), with atorvastatin, simvastatin, and rosuvastatin being the most prescribed due to their effectiveness in reducing cholesterol and cardiovascular risk. Alternative treatments like ezetimibe and PCSK9 inhibitors are used for statin-intolerant patients or those at high cardiovascular risk (Khan et al. 2021; Kim et al. 2022). Treatment decisions are based on factors such as efficacy, side effects, and liver function (Kulkarni et al. 2024). In addition to pharmacological intervention, lifestyle modifications, including smoking cessation, a healthy diet, and regular physical activity, are essential to preventing disease progression.

Genome-wide association studies (GWAS) of LDL-C have identified numerous genes involved in cholesterol regulation, including pathways related to LDL receptor activity, cholesterol biosynthesis, absorption, and lipoprotein metabolism. The GWAS catalog (Catalog 2024) compiles many LDL-C-associated genes, often showing small effect sizes. However, a significant proportion of genetic variability remains unexplained. This “missing heritability” is partly attributable to rare variants. Traditional GWAS, focused on common variants and failed to capture the impact of gene interactions or rare variants with large effects, limiting the development of precise individualised risk scores. Future research should explore novel gene-disease associations to advance personalised medicine.

In this study, we integrate genetic variant calls from rich whole exome sequencing using GenePy (Mossotto et al. 2019). GenePy is a tool to reduce the sparsity of variant calls files derived from genomic sequencing. It provides a whole-gene pathogenic burden score that aggregates all variants observed in a given gene in any one individual, into a single pathogenicity burden score for each gene. GenePy scores are intuitive whereby higher scores for any one gene reflects a higher pathogenic burden. Retaining signal from common *and* rare variation, GenePy has the potential to enhance causal disease gene detection and indicate genes with individually rare but collectively important clinical impact. This study differs from traditional association testing that detects altered genetic variant allele frequencies in cases compared to controls. Instead, we ask if the individuals with the highest pathogenic variant burden in a given gene are compared to those with the lowest pathogenic burden in the same gene, do they have significantly different blood cholesterol levels? We test this agnostically across all genes. The number of tests is limited by the number of genes in the genome and so controls for the ever-expanding set of rare and private variation observed as we sequence more individuals.

## Methods

### UK biobank

UK Biobank is a large-scale, prospective biomedical database containing phenotypic, lifestyle, clinical, imaging, and genetic data of 500,000 individuals. Consenting participants, aged 40–69 at the time of recruitment, were voluntarily recruited between 2006 and 2010 across the UK. The database is fully compliant with data privacy regulations and all records have been de-identified (Biobank 2024). Our study utilises the Phase 2 whole exome sequencing data of 200,620 individuals along with their respective phenotype and clinical data. UK Biobank access was provided under approved project ID 72911.

### Genetic data handling

To interpret variant-level data, we employed GenePy, a tool that quantifies the pathogenic burden of common and rare deleterious variation within individual genes (Mossotto et al. 2019; Rentzsch et al. 2019; Seaby et al. 2024). To calculate the GenePy score, all variants are annotated with deleteriousness, population frequency, and zygosity (indicating whether the variant is inherited from one or both parents) prior to aggregation of variant-level scores to generate gene-level scores for each individual.

All 10 million variants were annotated using CADD-Phred scores (CADD v1.6) (Rentzsch et al. 2019), which provide a normalised measure of deleteriousness for each variant (− 10 × log10 of the rank of a variant among 9 billion potential substitutions). Population allele frequencies were obtained from gnomAD (non-Finnish European population) (gnomAD 2022), using the Ensembl Variant Effect Predictor (VEP) (McLaren et al. 2016). We implemented a multi-step quality control and annotation pipeline to ensure the generation of a high-confidence variant dataset for subsequent analyses. Variants were initially filtered based on genotype quality, requiring a minimum read depth (DP *≥* 8) and allelic balance (AB *≥* 0.15) to retain reliable heterozygous calls. All homozygous reference genotypes (GT = “0/0”) were included in calculations. Variants with an F_MISSING value below 0.12 and Hardy-Weinberg Equilibrium (HWE) p-values exceeding the Bonferroni-corrected threshold of 0.05 were retained, ensuring a final dataset enriched for high-quality, biologically plausible variants with minimal genotyping errors. In order to prioritise variants with a higher likelihood of deleterious effects on gene function, we applied an additional filter to retain only variants with a CADD-Phred score *≥* 20. This filter can result in some genes where all individuals have a GenePy score of zero, indicating that the net effect of the variants within the gene was not deemed “pathogenic” by GenePy. Therefore, for downstream large data analysis, a cut-off threshold was applied to only select genes with more than five non-zero values, as tests on genes with fewer non-zero values are insufficiently powered. The final gene set excluded poorly annotated genes without International Commission on Genetic Nomenclature (ICGN) codes, Y chromosome genes, and olfactory genes (Karczewski et al. 2020).

To process scores for 20,031 genes across 200,620 individuals, we implemented GenePy (V3) [https://github.com/UoS-HGIG/GenePy-2/tree/V3/GenePy2_UKBiobank/Nextflow_Genepy2_UKBB_V3], leveraging the Nextflow framework (GenePy 2024) on a local high-performance computing cluster IRIDIS. Nextflow dynamically allocates CPU and RAM resources based on the specific requirements of each processing step, enabling scalable and efficient analysis of this large dataset.

### Clinical data handling

LDL-C measurement (UK Biobank field 30780) was taken as a continuous trait for analyses. For participants with multiple LDL-C measurements, the earliest measurement was taken. Participants with missing LDL-C values or values flagged as non-reproducible were excluded. Due to the known correlation whereby blood cholesterol levels increase with age in a manner independent of genetic mutation (Bertolotti et al. 2023), we analysed participants whose cholesterol measurements were taken when aged < 60 years separately from those whose cholesterol measurements were taken aged ≥ 60 years. Males and females were assessed separately for X-linked genes.

UK Biobank data include an estimate of kinship for each pair of individuals computed using KING software (Manichaikul et al. 2010). For this analysis, where individuals with third- or higher degree relationship were identified, we retained only the youngest participant to avoid potential confounding caused by related individuals. Age at the time of blood LDL measurement was calculated as the difference between their year of birth (field 34) and the date of the LDL assay (field 30781).

In addition to statins, drugs such as niacin (McKenney 2004), conjugated oestrogens, oestrogen products, and gestrinone (Feingold et al. 2017) can either increase or decrease LDL-C levels. Treatment/medication data (field 20003) was extracted for each participant (refer Supplementary Table S1 for full list of drugs). To avoid noise introduced by therapeutic alteration of LDL-C levels, participants on these drugs were excluded from downstream analyses.

### Statistical approaches used in our analysis

Using Python 3.9, we modelled blood LDL-C distributions across participant subsets. Shapiro-Wilk test was used to assess blood LDL-C normality. The Kolmogorov–Smirnov (K–S) test was used to compare blood LDL-C distributions between participant subsets: those not using cholesterol-lowering medications, those for whom medication usage data was unavailable, and those confirmed to be taking LDL-C-lowering drugs. The Mann-Whitney U test was employed to assess differences in blood LDL-C levels between participants with extreme GenePy values.

The preprocessed clinical and genomic data were integrated using participant ID. For each of the genes in the GenePy matrix, participants were ranked and binned, based on ascending GenePy scores, into 100 bins, with 1% of participants in each bin. Prior to statistical testing, the highest ranked percentile bin was checked and individual samples with GenePy scores of zero were excluded. Mann-Whitney U test was performed to compare LDL-C levels of participants in the lowest and highest ranked GenePy bins for each gene.

Due to the established sparse nature of genomic data, many of the lowest percentile GenePy bins were uniformly zero and individual assignment to these bins was arbitrary. Therefore, we bootstrapped the Mann-Whitney U test 1000 times, randomly shuffling participants each time before ranking in order of ascending GenePy score. For each gene, the mean p-value along with the standard deviation on 1000 iterations was recorded. Genes whereby a significant difference in blood LDL-C levels between participants with extreme quantile GenePy scores for any given gene, were adjusted using the Benjamini–Hochberg method (false discovery rate (FDR)). An FDR-adjusted p-value below 0.01 was taken as significant evidence of genetic impact on LDL-C levels.

KEGG 2021 human gene set (Kanehisa et al. 2025), which provides information on biological pathways, molecular interactions, and reaction network information, was applied using Enrichr in Python to identify overrepresented pathways among genes significant after FDR correction.

Protein-protein interaction networks depict the functional and physical interaction between a set of proteins and can be used to infer function, disease associations, and identify functional modules. We performed a network analysis of significant genes after FDR correction using the multiple proteins search facility provided by STRING-db website (Consortium 2024). Constraints were set to include interactions from very reliable sources only (experimental data and curated databases).

## Results

This section presents the outcomes of data preprocessing steps, the identification of significant genes associated with blood LDL-C levels, pathway enrichment analyses of the identified genes, and a protein-protein interaction (PPI) network analysis of the significant genes.

### Study population and data filtering

**Fig. 1:**
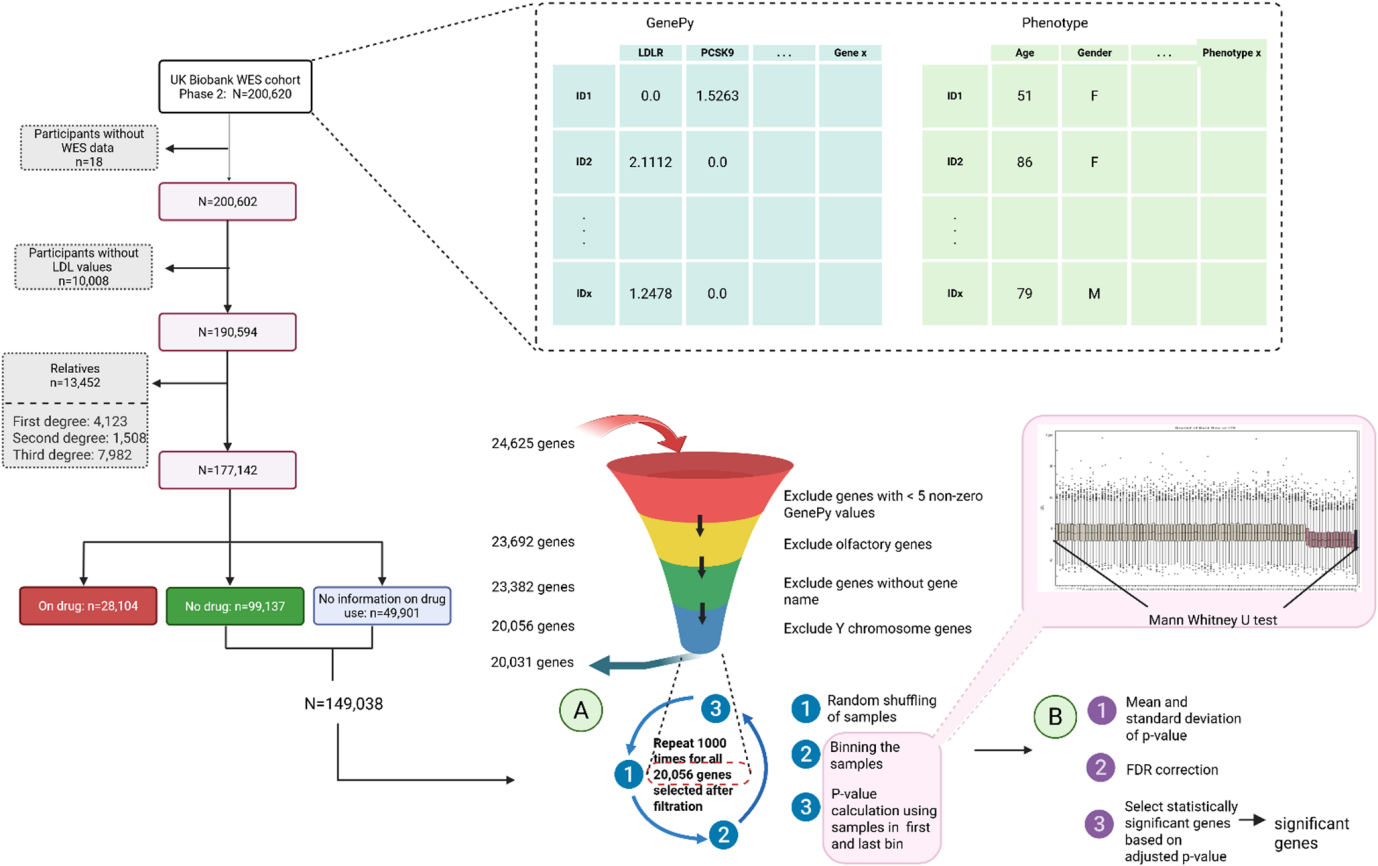
Workflow illustrating the preprocessing steps and statistical analysis pipeline

The initial cohort comprised 200,620 participants. Following data preprocessing, 18 participants without whole exome sequencing data, 10,008 participants missing LDL-C measurements, and 13,452 individuals identified as having at least third-degree relationships were excluded. A total of 177,142 participants were retained and stratified based on medication use into three groups: 99,137 participants not using any of the specified medications, 28,104 participants taking at least one drug impacting cholesterol, and 49,901 participants with no information on medication use (Fig. 1).

### LDL-C distribution and medication use

**Fig. 2:**
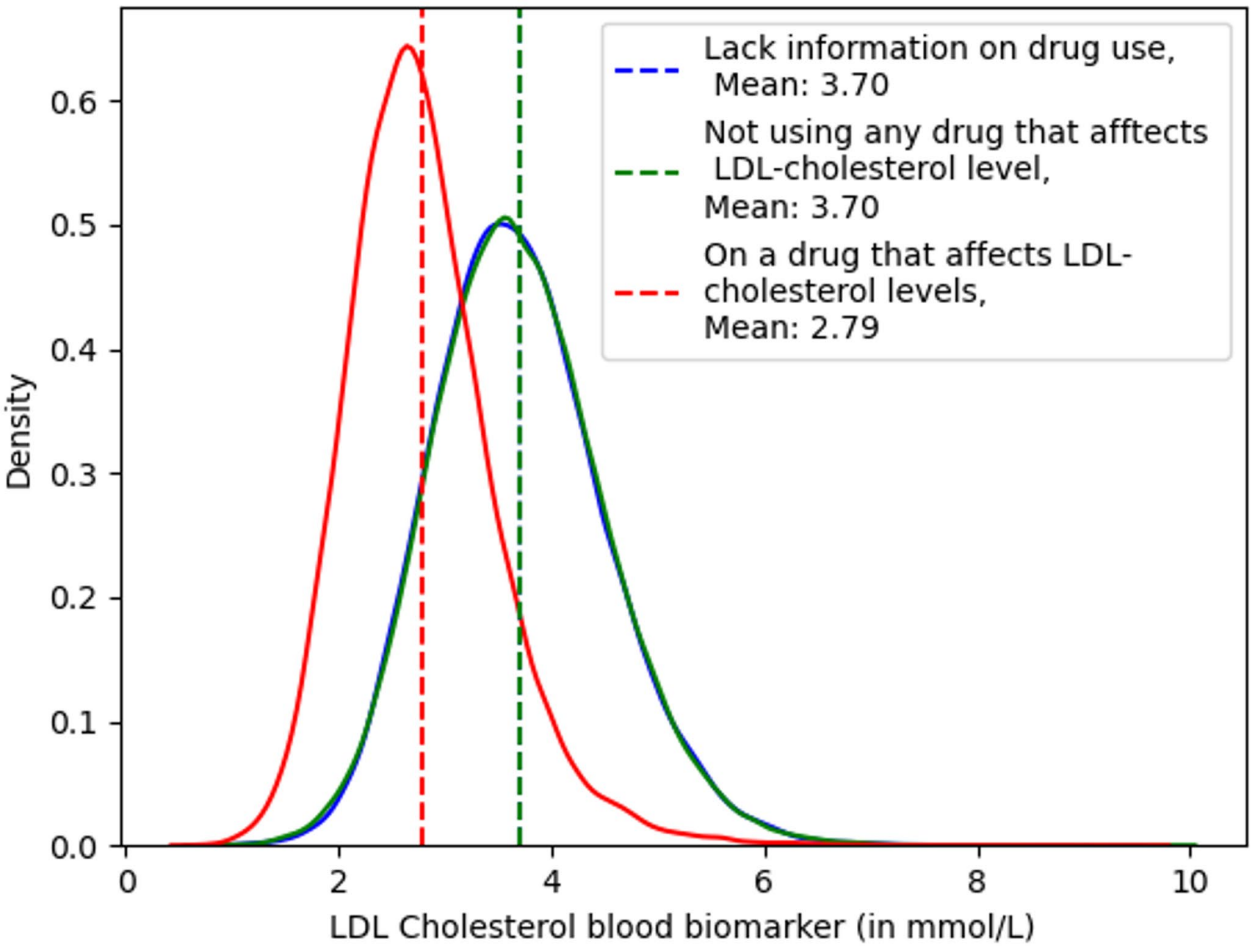
Density distribution of LDL-C values based on drug use information of participants

The density distribution of LDL-C levels across the above groups is presented in Fig. 2. Participants not taking any medications exhibited a mean LDL-C value of 3.70. The LDL-C density profile for participants with missing drug information did not significantly deviate from that of participants positively identified as *not* taking lipid lowering medication (K–S *p* = 0.29). This evidenced the assumption that those participants without evidence of relevant drug use were not administered lipid lower medication, and the groups were merged in subsequent analyses. As expected, the density distribution for participants reported on cholesterol lowering medication had significantly lower mean LDL-C (2.79 mmol/L) and these individuals were excluded from downstream analyses.

### Demographic characteristics

Demographic data for participants stratified by drug use is presented in Table 1. While the overall number of female participants exceeds that of males, the proportion of male participants on medication was higher, consistent with established trends indicating a higher prevalence of hypercholesterolemia and CVD among men (Ingelsson et al. 2007). Additionally, medication use increased with advancing age, highlighting the link between age and the need for pharmacological interventions. Among participants on drug, women exhibit a higher mean and standard deviation in LDL-C compared to men.

### Gene set refinement

The initial dataset included 24,625 genes. Following exclusion of genes with fewer than five nonzero values (*n* = 933), olfactory genes (*n* = 359), poorly annotated genes (*n* = 3277), and Y chromosome genes (*n* = 25)-a final set of 20,031 genes were assessed in 149,038. Autosomal genes (*n* = 19,270) were assessed separately to X chromosome genes.

**Table 1:** Clinical characteristics of UK biobank participants used for analysis. Data are presented for two categories of participants. The “not on drug” category merges individuals who are not taking any cholesterol-lowering medications as well as those for whom information on medication use is unavailable

Demographics	Not on drug (*N* = 149,038)			On drug (*N* = 28,104)			All
	Female (*n* = 86,244)	Male (*n* = 62,724)	All	Female (*n* = 11,123)	Male (*n* = 16,981)	All	
Age in years							
< 60	33.33%	34.75%	50,563	07.63%	09.11%	2,396	97,367
* ≥* 60	66.65%	65.24%	98,443	92.29%	90.85%	25,692	79,775
Smoking							
Never smoked (0)	60.98%	52.25%	85,399	55.06%	38.55%	12,670	98,069
Former smoker (1)	31.08%	35.44%	48,672	35.52%	49.46%	12,350	61,022
Current smoker (2)	07.94%	11.86%	14,291	08.65%	11.27%	2875	17,166
BMI in kg/m^2^							
Mean:	26.67	27.40	26.98	29.41	29.25	29.32	27.35
Range:	56.83	53.53	56.83	49.78	42.47	49.78	56.83
Std:	04.99	04.03	04.62	05.61	04.46	04.95	04.75
LDL-C in mmol/L							
Mean:	03.71	03.70	03.70	02.90	02.71	02.79	03.56
Range:	08.99	08.03	08.99	08.71	06.35	08.82	09.09
Std:	00.84	00.78	00.81	00.72	00.68	00.70	00.86

### Genetic links to LDL-C

**Fig. 3:**
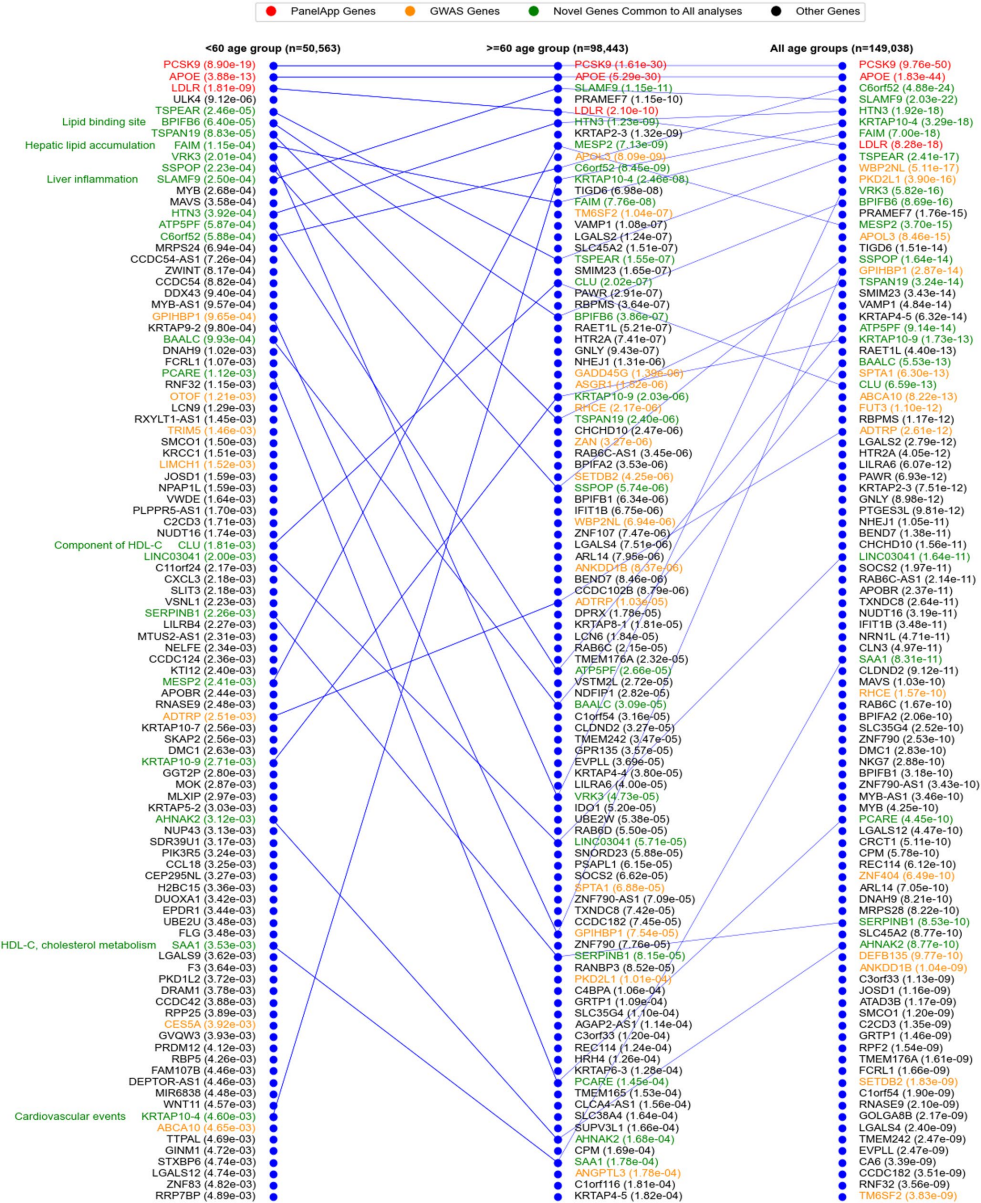
Parallel coordinate plot displaying the 100 genes with most significant difference in pathogenic burden comparing patients with lowest and highest blood cholesterol levels. Results for participants whose blood cholesterol measurement was recorded at an age < 60 years (*n* = 50,563, left panel), ≥ 60 years (*n* = 98,443, middle panel) and finally all participants (*n* = 149,038, right panel). Genes are colour-coded to indicate PanelApp genes that are routinely tested following referral for (familial) hypercholesterolemia (red); genes previously identified through GWAS (orange); novel genes not previously implicated in GWAS that are common to subgroups (green). Novel genes are annotated with keywords indicating evidence for potential plausible function in influencing blood cholesterol. Nominal p-values are shown for all genes. *HDL-C* HDL cholesterol

GenePy scores were calculated for all individuals and for each gene, individuals were ranked by their score and allocated to percentile bins. Mann Whitney U tests were conducted by comparing LDL-C levels between the lowest and highest ranked GenePy score bins for each gene. This analysis was conducted for participants whose LDL-C levels were measured at an age < 60 years (n_< 60 years_=50,563) and ≥ 60 years (n_≥60 years_=98,443); and for the combined group (n_all_=149,038). Thirty-two participants with missing age information were included exclusively in the combined analysis. Analysis limited to the younger group identified 665 nominally significant (*p* < 0.05) autosomal genes, with only three genes achieving FDR significance. Analysis of group measured aged ≥ 60 years, 1,066 autosomal genes were nominally significant with 56 genes withstanding FDR adjustment. The combined group analysis identified 2,514 genes as nominally significant, of which 668 were significant after FDR correction. A parallel coordinate plot shows the top 100 genes across all three analyses (Fig. 3). Our study successfully identified established genes known to causally influence LDL-C levels. The genes with most significant difference in LDL-C levels in individuals with the highest GenePy pathogenic burden versus those with the lowest, were those routinely used in clinical practice and the NHS Genomic Test Directory PanelApp(England 2023) for hypercholesterolemia. Of the five PanelApp genes indicated for diagnostic assessment, three (*PCSK9*,* APOE*, and *LDLR*) are amongst those for whom individuals with the most extreme GenePy scores, have the greatest difference in blood cholesterol levels in all analyses (Fig. 3). This result reinforces both the critical role of these genes in lipid metabolism at the population level and the sensitivity of our analytical approach using GenePy. A fourth PanelApp gene, *APOB*, was significant in the analysis of LDL-C measurement taken at any age (*p* = 1.6 × 10^−4^) and in those age ≥ 60 years (*p* = 9.02 × 10^−4^), but cholesterol measurements were not significantly different in individuals with lowest and highest pathogenic burden in APOB in the smaller group where the measurement was taken aged < 60 years. Our approach did not identify the fifth gene, *LDLRAP1*, likely due to the extreme rarity with which this gene impacts cholesterol across populations.

**Fig. 4:**
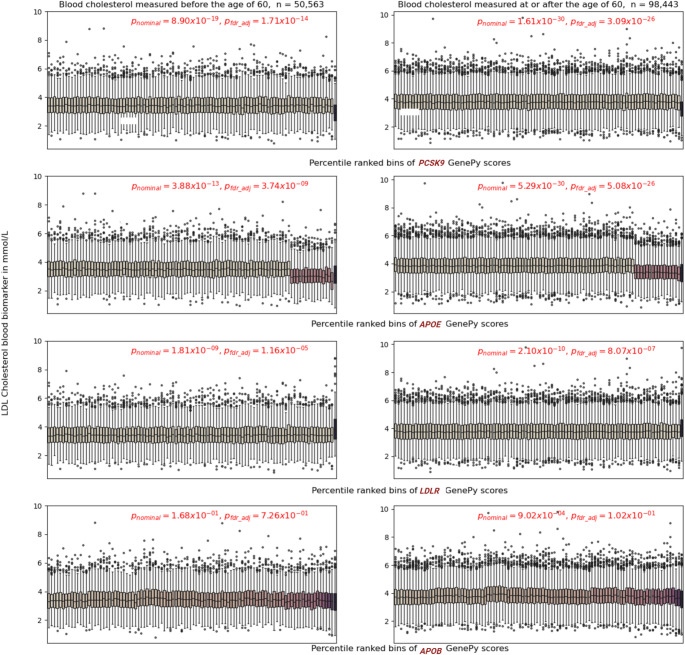
Histogram shows the relationship between LDL-C and GenePy scores of UK Biobank participants across 100 percentile bins. All p-values are embedded in the figure. **Left panel**: LDL-C measurements recorded at an age < 60 years; **Right panel**: LDL-C measurements recorded at an age ≥ 60 years

We considered the GenePy percentile distribution for the diagnostic PanelApp genes across blood cholesterol measurements taken at an age < 60 years and ≥ 60 years (Fig. 4). For both groups, a distinct shift in LDL-C values in the final bin of *LDLR*, is observed. Individuals ranked in the highest percentile of GenePy scores for *LDLR*, had a markedly higher blood cholesterol level compared to those in the lowest bin (p_< 60 years_=1.16 × 10^−5,^ p_≥60 years_=8.07 × 10^−7^). This is consistent with the expectation that pathogenic LoF variants in the gene encoding the LDL receptor protein, results in higher circulating cholesterol levels. Somewhat counterintuitively, we observed a *protective* effect of the *PCSK9* gene showing that across a large population, *LoF* variants in this gene impose a cholesterol *lowering* effect and this outweighs the known, but very rare *gain of function* variation very infrequently observed in families with FH. The pattern for *APOE* is distinctive, whereby much more common variation that influences pathogenic mutation burden evident across the top 16 bins has a significant cholesterol lowering impact across cholesterol measurements taken at age ≥ 60 and age < 60 years (p_≥60 years_=5.08 × 10^−26^, p_< 60 years_=3.74 × 10^−9^). In the most extreme bins however, this effect is tempered by genetic variation with opposing effect. These data underpin the unmet need to better characterise the direction of individual variant effects. *APOB* is a gene characterised by much more extensive genetic heterogeneity across the whole cohort. For this gene, most individuals have a GenePy score > 0, and we observe a gradual but significant (*p* = 9.02 × 10^−4^) decline in blood cholesterol in individuals ranked in the highest percentile for pathogenic burden when analysis is done using ≥ 60 years group. We do not observe a significant difference in cholesterol levels comparing individual at extreme percentiles for this gene in < 60 years group.

In addition to implicating known diagnostics disease genes, our analyses detected genes previously identified through GWAS (Fig. 3). Furthermore, significant signals were observed in genes not previously associated with blood LDL-C. Interestingly, a number of these genes confer function with plausible relevance to lipid handling. These findings demonstrate potential of alternative methods that encompass rare variation in identifying potentially new contributors to LDL-C regulation.

The analysis of the X chromosome genes provided some insights. Insulin receptor substrate 4 (*IRS4*) gene withstood FDR correction (*p* = 6.91 × 10^−3^) in the analysis that considers cholesterol measurements taken from male participants who were ≥ 60 years (Supplementary Figure S3). It is nominally significant (*p* = 1.02 × 10^−2^) in the analysis of cholesterol levels of females in the same age group. Isocitric dehydrogenase subunit gamma (*IDH3G*), a gene involved in carbohydrate metabolic process, is another notable gene appearing in the top list of both these analyses (p_female_=1.96 × 10^−4^, p_male_=5.09 × 10^−3^).

### Pathway enrichment analysis of LDL-C

Genes significant in the analysis of blood cholesterol measured at any age (n_FDR corrected_=668) were aggregated based on pathway information, enabling the assessment of enrichment of significant genes within molecular functions. Table 2 identifies six enriched pathways, with roles in lipid metabolism being well-supported by existing literature(Gudas 2022). The metabolism of xenobiotics by cytochrome P450 represents the most significantly enriched pathway (*p* = 3.79 × 10^−3^). Unsurprisingly, the cholesterol metabolism pathway governing synthesis, transport, and regulation of cholesterol levels, was identified as highly significantly enriched in the genes implicated in blood cholesterol measurements at any age, in age ≥ 60 years, and in age < 60 years (p_all_=6.19 × 10^*−*3^, p_≥60_=3.71 × 10^−4^, and p_< 60_=1.47 × 10^−8^) (Supplementary Table S2, Table S3). The steroid hormone biosynthesis pathway (*p* = 1.69 × 10^*−*2^) facilitates the enzymatic conversion of cholesterol into various steroid hormones. The ABC transporter pathway (*p* = 1.75 × 10^*−*2^) is essential for the transport of diverse substrates, including lipids and cholesterol trafficking. Together, these findings underscore the multifaceted genetic regulation of cholesterol metabolism and transport and underpin the mechanisms influencing LDL-C levels. While a direct link between the pentose and glucuronate interconversions pathway and LDL-C metabolism is not well characterised, its involvement in carbohydrate metabolism(BioPortal 2024), which can influence lipid regulation, may indicate an indirect role.

### Network analysis

**Fig. 5:**
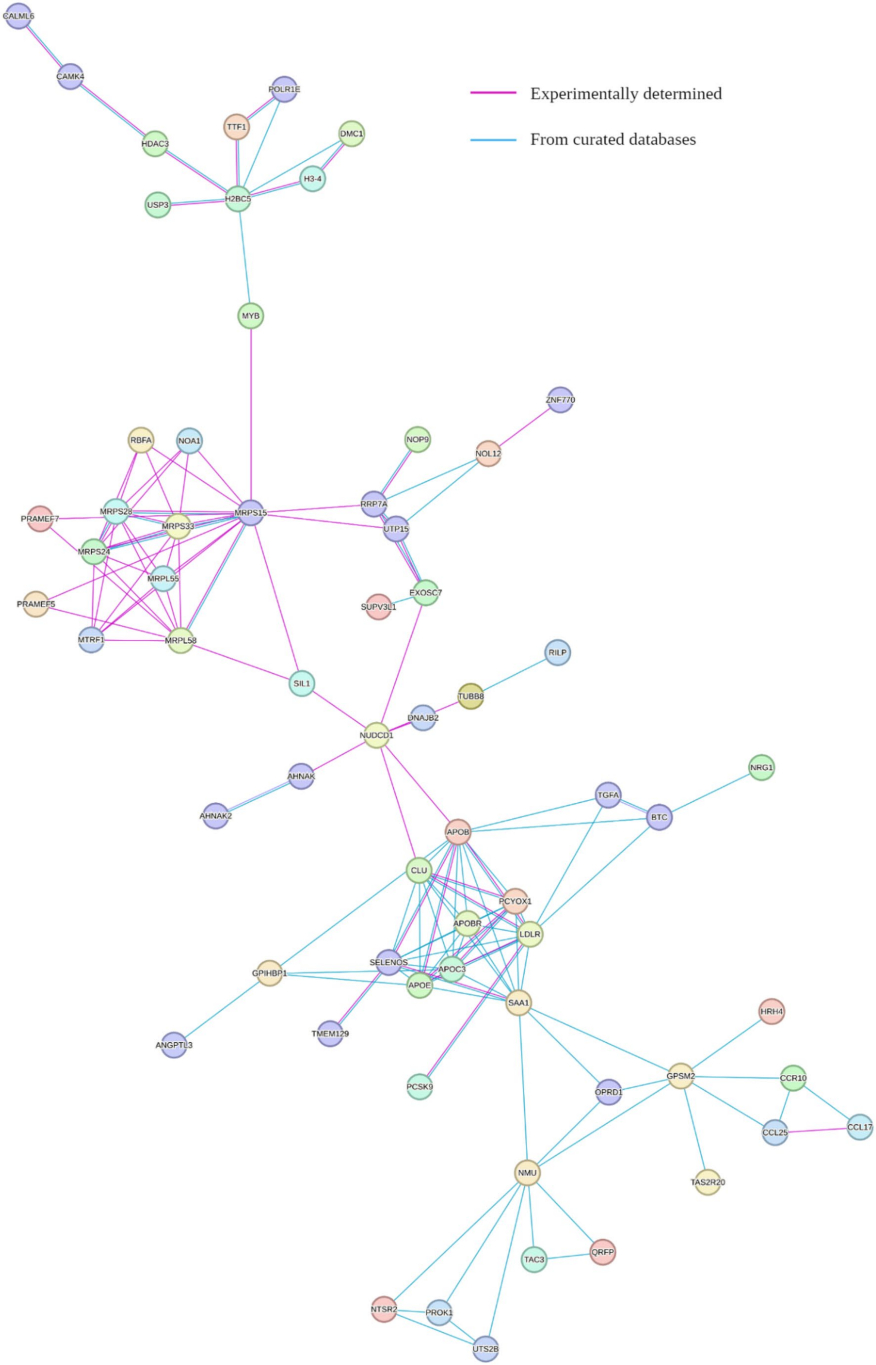
Protein–protein interaction network generated using STRING-db based on FDR-significant genes from “all participants” analysis. Each node represents a protein encoded by a significant gene, while edges indicate functional and physical associations. Magenta edges denote experimentally validated interactions, and teal blue edges represent interactions curated from databases. Node colorings are random and have no significance

Network analysis performed using STRING-db revealed functional and physical associations among proteins encoded by genes identified as FDR significant in the analysis involving cholesterol measurements taken at any age. These genes are depicted as nodes in the network, while edges indicate evidence-supported interactions (Supplementary Fig. S3). Figure 5 depicts the network interactions for the largest observed subgraph that connects a substantial number of genes identified by our analysis as biologically significant. Interestingly, proteins encoded by novel gene candidates such as *CLU*,* SAA1*, and *AHNAK2*, which rank among the top 100 genes in all analyses, are found to interact with well-established genes, including *APOE*,* LDLR*, and *PCSK9*. These interactions strengthen the potential relevance of these novel genes in influencing blood cholesterol.

**Table 2:** Enrichment analysis of FDR significant genes from analysis considering cholesterol measurements taken at any age

Pathway	*p*	Combined score	Genes
Metabolism of xenobiotics by cy- tochrome P450	3.79 × 10^*−*3^	19.14	*UGT2B10*,*AKR7 A3*,*CYP2B6*,*GSTA3*,* UGT2B15*,* DHDH*,* CYP3 A5*,* CBR3*
Cholesterol metabolism	6.18 × 10^*−*3^	20.20	*ANGPTL3*,* PCSK9*,* APOC3*,* APOE*,* APOB*,* LDLR*
Steroid hormone biosynthesis	1.59 × 10^*−*2^	13.15	*UGT2B10*,*CYP3 A7-CYP3 A51P*,* UGT2B15*,* AKR1 C4*,* CYP3 A5*,* CYP3 A7*
ABC transporters	1.66 × 10^*−*2^	14.90	*ABCA10*,* ABCB5*,* TAP1*,* ABCA7*,* ABCC12*
Retinol metabolism	2.58 × 10^*−*2^	10.30	*UGT2B10*,*CYP3 A7-CYP3 A51P*,* CYP2B6*,* UGT2B15*,* CYP3 A5*,* CYP3 A7*
Pentose and glucoronate interconver- sions	2.59 × 10^*−*2^	14.17	*UGT2B10*,* UGT2B15*,* DHDH*,* SORD*

The first column represents the enriched pathways

‘Combined score’ is a function of p-value and z-score, i.e., it integrates statistical significance and strength of enrichment

Only significant pathways are included in the table

## Discussion

The GenePy tool offers an alternative method for linking rich genetic variation with clinically relevant phenotypes. The score integrates rare variation overlooked by GWAS with common variants. It collapses the vast set of all variations observed through sequencing studies, into a pathogenic burden score for each gene, that can be compared between individuals. Integrating frequency and deleteriousness into the algorithm takes advantage of our improving resources and has the potential to detect novel associations not previously observed.

Encouragingly, our approach using GenePy score identifies the known genes of most important clinical significance amongst the most significant results. *PCSK9* is the gene with most significant change in cholesterol levels comparing individuals with the highest and lowest percentile scores when GenePy modelling incorporates the collective common impact of rare deleterious variants. Gain of function mutations in this gene are a known cause of familial hypercholesterolemia and individual variants conferring this effect are detected through diagnostic sequencing. However, our data convey the wider impact of loss of function (LoF) in this gene in *suppressing* LDL cholesterol at a population level. This protective effect warrants clinical consideration when assessing an individual patient’s personalised risk. Our investigation also identified the important FH diagnostic genes *APOE* and *LDLR* amongst the most highly significant. Our population level analysis did not detect the very rare but established role for the *LDLRAP1.* This is likely because our scan of all genes allocates individual into relatively broad percentile bins, and the proportion of individuals actually impacted by this gene is substantially less than 1%. Our aim in this study was to identify aggregated signals of variation whereby, although individual variants may be rare, their collective contribution to disease impacts at least 1% of the cohort. An alternative approach using GenePy that prioritised assessment of individuals with the most extreme scores has proven successful in detecting causes of very rare clinical manifestations(Seaby et al. 2024). Such an application was outside the scope of the current study.

In addition to strongly established causal genes, our study identified several additional genes linked to CVD, diabetes, metabolic syndrome, and neurological disorders in relation to LDL-C through analyses of entire cohorts, elder sub-cohorts and younger sub-cohorts. While a number of these genes were previously implicated by GWAS, we detected genes with strong plausible functional relationship to cholesterol handling that warrant further scrutiny. The significant association of these genes with LDL-C, observed in analyses using two independent sample sets from different age groups, underscores their potential importance.

### Implications of lipid associated genes in metabolic regulation, blood pressure, and lipid traits

Our analysis identified several genes implicated in lipid-associated traits, including fat metabolism and lipid accumulation. Although no direct association between *BPIFB6* and LDL-C has been previously reported, this gene was identified in both independent groups of individuals with cholesterol measurements taken pre and post 60 years. This gene is not well studied but is structurally related to proteins capable of binding phospholipids and lipopolysaccharides (Mulero et al. 2002). *FAIM* encodes a protein regulator of pituitary adenylate cyclase-activating polypeptide (PACAP) that has an important metabolic role in attenuating hepatic lipid accumulation, obesity-induced insulin resistance (Feng et al. 2024) and lipid metabolism in obese liver (Xiao et al. 2019) to attenuate metabolic disorders by reducing hepatic lipid accumulation (Luo et al. 2022). Results across the independent pre and post 60 cholesterol measurement subgroups replicate an effect of *SAA1* gene. Studies suggest *SAA1* has critical relationship with HDL-C level (Carty et al. 2009), can potentially alter lipid homeostasis (Sullivan et al. 2010), has regulatory function in cholesterol metabolism (Huang et al. 2024), and its suppression can help in high fat diet induced insulin resistance (Wang et al. 2019). *CLU* in its secreted form is a component of HDL-C and has role in metabolic and cardiovascular diseases (Park et al. 2014). While the *APOB* gene is firmly established in FH, our results implicate its receptor *APOBR* and it is perhaps unsurprising that the collective impact of rare and common variation in this gene alters cholesterol (Fujita et al. 2005). The gene shows nominal significance in the analyses of both age groups, and in the combined analysis, it withstood FDR correction (p_FDR_adj_=9.72 × 10^−9^).

*ANGPTL3* is a known GWAS gene linked to LDL-C, with published studies confirming its role in cardiovascular events in older populations (Hussain et al. 2021). Our findings align with this, as the gene shows significant associations (*p* = 1.78 × 10^−4^) in older cohort and does not demonstrate any significance in younger population.

*IRS4* is part of insulin signaling pathway and its upregulation can lead to insulin resistance (Pandey et al. 2023). *IDH3G* is of importance because of its involvement in the peroxisomal lipid metabolism superpathway. A reduced expression of this gene is found in patients with arteriosclerosis and abdominal aortic aneurysm (Gu et al. 2024).

### Liver enzyme regulation and genetic links with LDL-C levels

The liver is the regulatory hub for serum cholesterol levels and hepatic dysfunction is known to impact LDL-C levels (Chrostek et al. 2014; Jiang et al. 2014). The combined activation of *SLAMF9* and *SLAMF8* induces macrophage activity, while their downregulation modulates the expression of *TLR4*, thereby attenuating endotoxin-induced liver inflammation (Zeng et al. 2020). Experiments conducted on two strains of mouse implicated the protein encoded *SLAMF9* in cholesterol loading (Berisha et al. 2013). Our findings add further evidence implicating this gene with blood cholesterol measured at any age.

### Genetic insights into cardiovascular diseases and associated traits

Our study has identified several genes associated with cardiovascular diseases. GWAS have previously linked the Keratin Associated Protein 10-4 (*KRTAP10-4*) gene with increased risk of major adverse cardiovascular events (MACE) (Liu et al. 2021). Specifically, the missense variant *rs201441480* in *KRTAP10-4* has been identified as a potential risk factor for MACE. Although the underlying mechanisms remain unclear, our study now adds to the evidence suggesting that variation in this gene may influence LDL-C levels.

### Limitations

Volunteer-based recruitment of participants to the UK Biobank cohort used in this study imposes limitations on the generalisability of the findings to a broader population. Over 90% of the participants are of European ancestry, restricting the applicability of results to other ethnic groups. Additionally, only 5.5% of the UK population at the time was represented in the study, with a majority being older adults, women, and individuals from higher socio-economic strata(van Alten et al. 2024). UK Biobank is a globally recognised resource of considerable value to the research community, but there are some limitations to the depth of clinical data and the rigour of self-reported data.

The GenePy framework has inherent limitations in common with many statistical genetic approaches. While GenePy scores can be tuned to integrate only variants inferred to have functional impact (CADD scores > 20), this does not account for the directionality of the effect. Although protein truncating and splice variants can be assumed to cause LoF, missense variants are far commoner and the research community lacks reliable inference of gain or loss of function for most variants in this important class.

In our study, we used CADD 1.6 to infer variant deleteriousness for incorporation into the GenePy score. No in silico predictor of variant deleteriousness is infallible and there are likely limitations to CADD, however this score applies a balanced approach to sense and missense coding variants as well as all non-coding variation– a feature lacking in many other deleteriousness annotation tools. Common to all analyses using genomic data derived from short read sequencing, the variants called from these data are unphased– meaning they are not reliably assigned to either the maternal or paternal haplotype. Efforts to estimate phase fail for rare variation. Therefore, the GenePy scores generated herein, represent a composite score reflecting the combined burden from both maternal and paternal chromosomes and this obfuscates genetic signal and reduces power to link variation to phenotypes. As the costs of long-range sequencing falls, the ability to calculate GenePy scores for each parental copy will afford more refined modelling and greater sensitivity for signal detection.

## Conclusion

Genes identified in previous genetic associations of hypercholesterolemia, are unable to explain all the genetic pathogenicity of the disease. Alternative approaches that make improved use of the vast amounts of rare variant data observed through sequencing are likely to identify at least some of this missing heritability. Our study, which uses the GenePy score, demonstrates sensitivity in recapitulating the strongest known genetic causes. The approach further uncovers strong evidence suggesting additional genes in functional pathways established to be critical in cholesterol homeostasis. These genes may harbour rare variation that collectively impact a clinically relevant fraction of the UK population. It is desirable that we move towards a model of prediction and early intervention in order to reduce the health economic burden that is the sequelae of raised cholesterol. However, to be effective, approaches using polygenic risks scores demand that variant data on the most comprehensive set of genes that alter cholesterol handling are inclusively modelled. Our study suggests the need to consider a wider set of genes that may harbour rare variation impacting the common phenotype of raised cholesterol and suggests the need for further independent studies across different populations.

## Supplementary Information

Below is the link to the electronic supplementary material.

Supplementary file1 (DOCX 877 KB)

Supplementary file1 (DOCX 2947 KB)

## Data Availability

GenePy data used in this work is available at https://github.com/UoS-HGIG/GenePy-2/tree/V3/GenePy2_UKBiobank/Nextflow_Genepy2_UKBB_V3.
