# Supplementary material for: Identification of genetic biomarkers of blood cholesterol levels using whole gene pathogenicity modelling
Source: Mamm Genome. 2025 Jun 6;36(3):914–27. doi: 10.1007/s00335-025-10140-0 (PMC12408753; doi:10.1007/s00335-025-10140-0)
Supplement: Supplementary file 1 — Supplementary file1 (DOCX 877 KB) [file 335_2025_10140_MOESM1_ESM.docx]

# Medication codes

**Table S1.** Details of medications that can alter LDL-C levels

| Code | Drug |
| --- | --- |
| 1140881748 | zocor 10mg tablet |
| 1140861958 | simvastatin |
| 1140888594 | fluvastatin |
| 1140864592 | lescol 20mg capsule |
| 1140888648 | pravastatin |
| 1141146234 | atorvastatin |
| 1141192410 | rosuvastatin |
| 1141192414 | crestor 10mg tablet |
| 1141188146 | simvador 10mg tablet |
| 1141192736 | ezetimibe |
| 1140861954 | fenofibrate |
| 1140862026 | ciprofibrate |
| 1140861944 | clofibrate |

# Statistical tests for normality of LDL-C values

Although the density plot in Figure 2 in main text appears approximately bell-shaped, the Shapiro-Wilk test indicated that the LDL-C values satisfy the assumption of homoscedasticity (p-value = 0.27) but deviate from normality (p-value = 0.0000). Hence the choice of Mann-Whitney U test.

# Handling relationship data

Generate a weighted relationship network, as shown in Figure S[1](#_bookmark0), with each individual as a node, the relationship between them as edges, and the kinship coefficient as the weight of the edge. Each node and all nodes connected to this node, are treated as a group, provided that the kinship coefficient between the initial nodes and connected nodes are greater than 0.0442[1] (the lower limit of kinship coefficient for a third-degree relationship). The youngest individual within a grouping is retained for analysis, while all other individuals in the group are excluded. The procedure is repeated for all remaining nodes in the network. A sample network is given in Figure S[1](#_bookmark0).


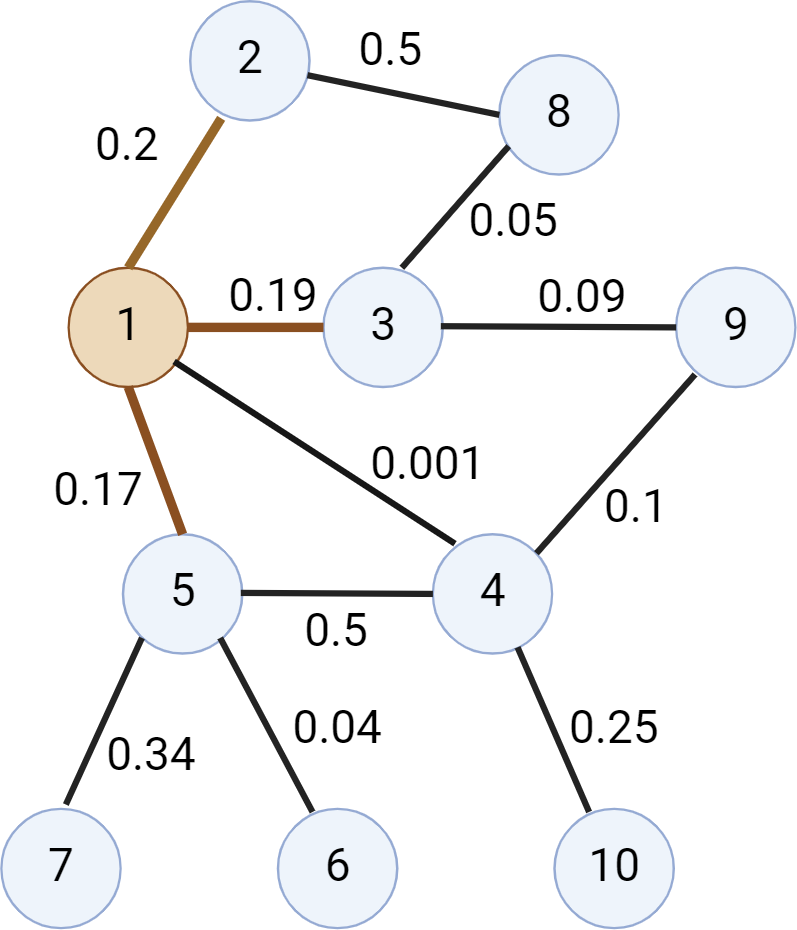


**Figure S1.** An example weighted relationship network of participants in the UK Biobank, where the weights represent the kinship coefficient. Genetic relatives of individual ’1’ who are related by at least third degree are depicted as nodes connected to individual ’1’ through brown lines.

# 4. Gene Enrichment Analysis of Significant Genes

Gene set enrichment analysis on the significant genes (n = 3) identified by the analysis that used data on participants age <60 at time of cholesterol measurement (Table S2) highlighted a significant role for the cholesterol metabolism pathway (p = 1.47 x 10^-8^), specifically involving *PCSK9, APOE*, and *LDLR*. Additionally, pathways closely associated with *LDLR*, including ovarian steroidogenesis, cortisol synthesis and secretion, bile secretion, and aldosterone synthesis and secretion, were found to be of particular relevance in this subgroup. This indicates a potential age-dependent distinction in the genetic pathways influencing LDL-C levels, with *LDLR*-related mechanisms playing a more prominent role in younger individuals. These findings underscore the complexity of cholesterol regulation and the need to consider demographic-specific genetic contributions.

**Table S2.** Enrichment analysis of FDR significant genes from analysis that considers cholesterol measurements taken at age<60 years. The first column represents the enriched pathways. ‘Combined score’ is a function of p-value and z-score, i.e., it integrates statistical significance and strength of enrichment. Top 10 significant pathways are included in the table.

| Pathway | p | Combined score | Genes |
| --- | --- | --- | --- |
| Cholesterol metabolism | 1.47x10*^−^*^8^ | 1.08 x 10^6^ | *PCSK9, APOE, LDLR* |
| Ovarian steroidogenesis | 7.63x10*^−^*^3^ | 972.53 | *LDLR* |
| Cortisol synthesis and secretion | 9.72x10*^−^*^3^ | 721.59 | *LDLR* |
| Bile secretion | 1.34x10*^−^*^2^ | 481.99 | *LDLR* |
| Aldosterone synthesis and secretion | 1.46x10*^−^*^2^ | 433.37 | *LDLR* |
| Toxoplasmosis | 1.67x10*^−^*^2^ | 366.54 | *LDLR* |
| Cushing syndrome | 2.31x10*^−^*^2^ | 242.83 | *LDLR* |
| Hepatitis C | 2.34x10*^−^*^2^ | 238.88 | *LDLR* |
| Lipid and atherosclerosis | 3.19x10*^−^*^2^ | 159.23 | *LDLR* |
| Endocytosis | 3.73x10*^−^*^2^ | 129.33 | *LDLR* |

**Table S3.** Enrichment analysis of FDR significant genes from analysis that considers cholesterol measurements taken at age≥60 years. The first column represents the enriched pathways. ‘Combined score’ is a function of p-value and z-score, i.e., it integrates statistical significance and strength of enrichment.

| Pathway | p | Combined score | Genes |
| --- | --- | --- | --- |
| Cholesterol metabolism | 3.71x10*^−^*^4^ | 1.89 x 10^2^ | *PCSK9, APOE, LDLR* |
| Non-homologous end-joining | 3.58x10*^−^*^2^ | 1.00 x 10^2^ | *NHEJ1* |

# References

1. Manichaikul A, Mychaleckyj JC, Rich SS *et al.* Robust relationship inference in genome-wide association studies. *Bioinformatics* 2010;**26**:2867–73.


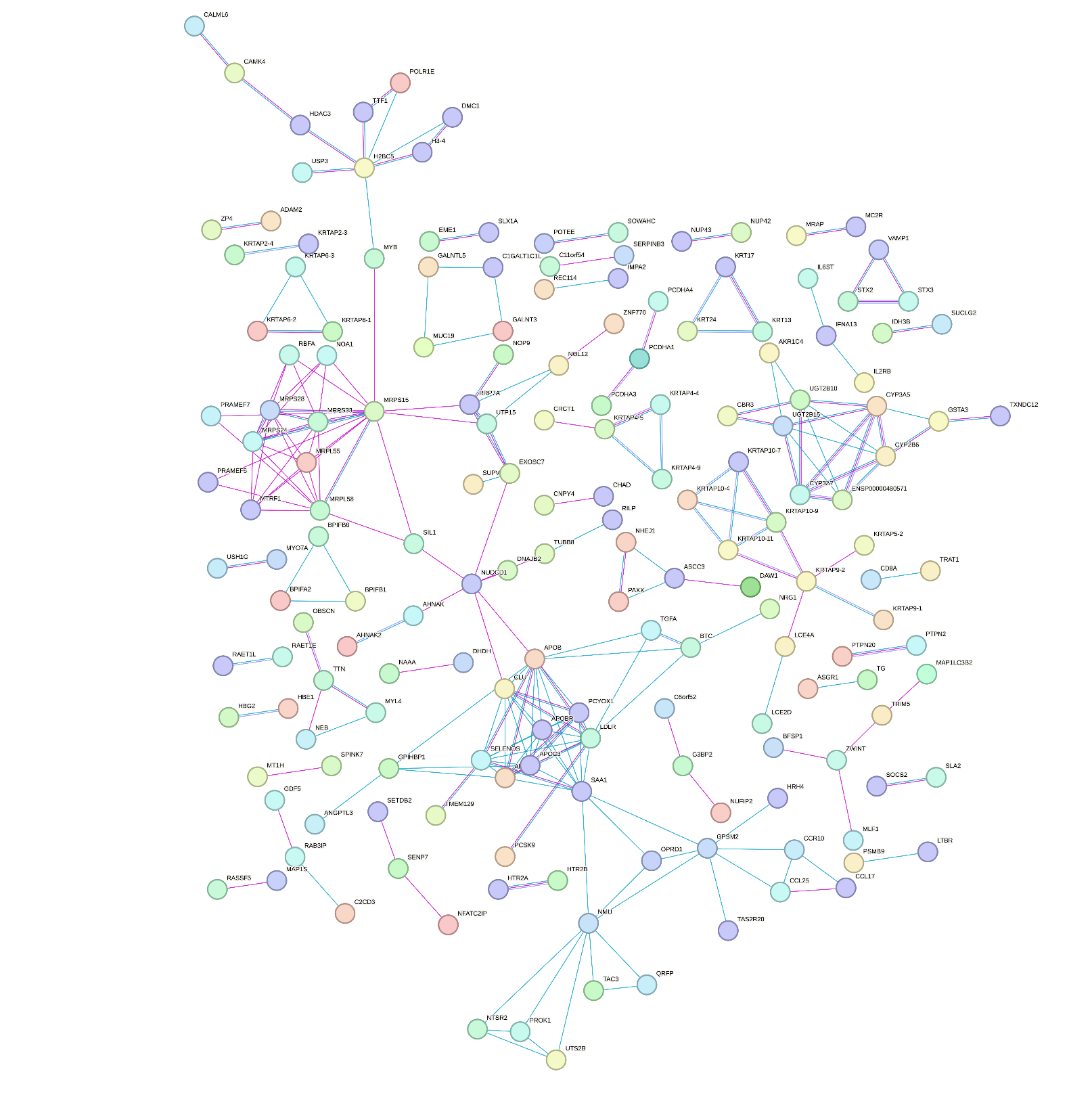
**Figure S2.** Protein-protein interaction network generated using STRING-db based on FDR-significant genes from analysis considering LDL-C measurements taken at any age. Each node represents a protein encoded by a significant gene, while edges indicate functional and physical associations. Magenta edges denote experimentally validated interactions, and teal blue edges represent interactions curated from databases. Node colorings are random and have no significance. Disconnected components are hidden in this network


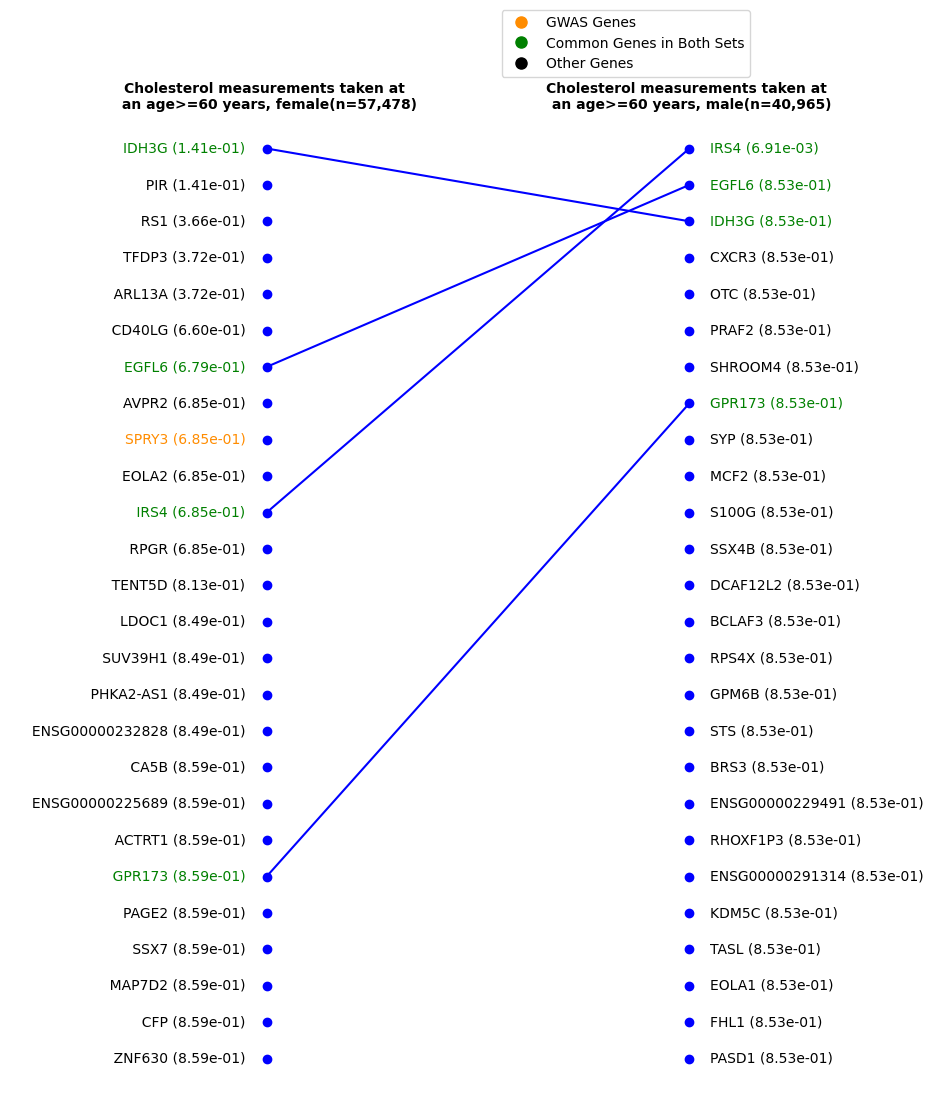


**Figure S3.** Parallel coordinate plot showing top 25 genes from analyses that uses cholesterol measurements taken at or later than 60 years for female and male groups. FDR adjusted values are given with each gene.


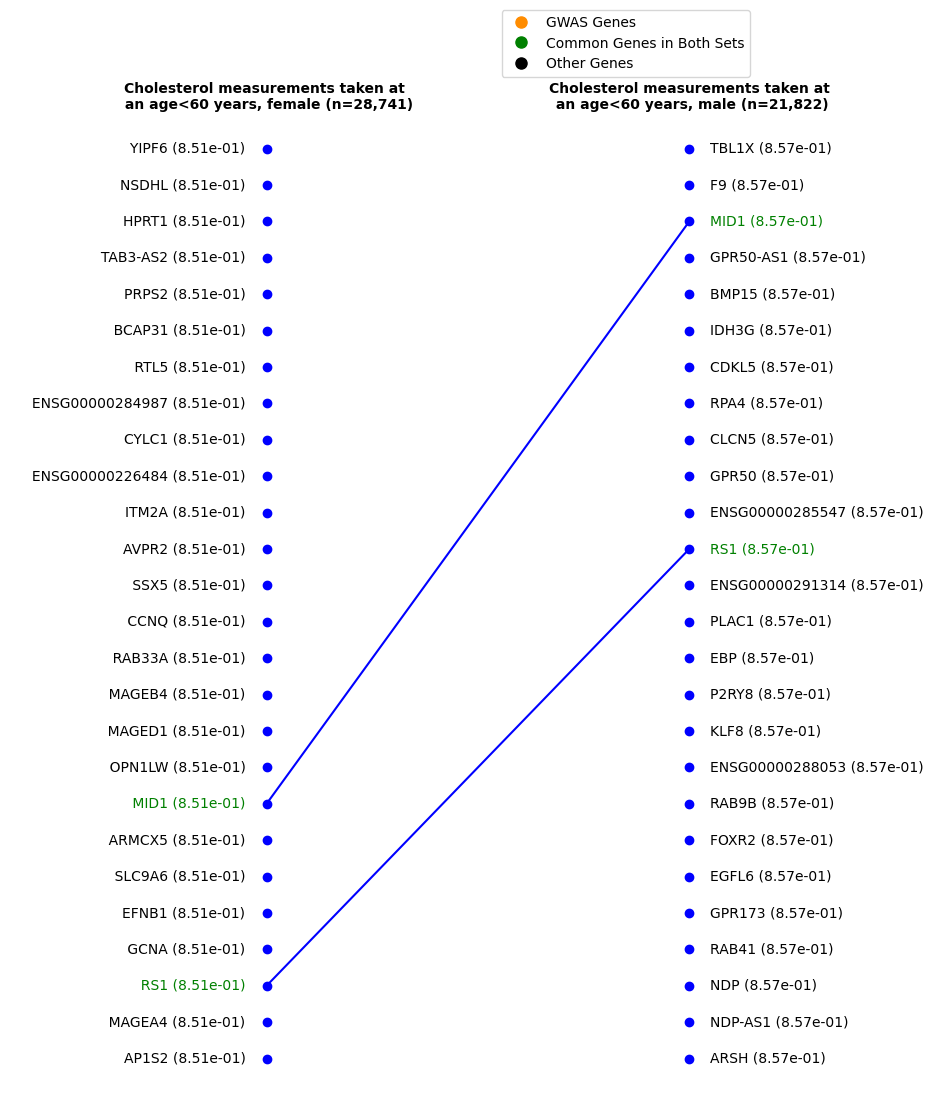


**Figure S4.** Parallel coordinate plot showing top 25 genes from analyses that uses cholesterol measurements taken at an age< 60 years for female and male groups. FDR adjusted values are given with each gene.
